# Supplementary material for: Innovative population-based strategies for primary prevention of cardiovascular disease: A 2-year randomised control trial evaluating behavioral change led by community champions versus brief advice
Source: PLoS One. 2024 Dec 18;19(12):e0314748. doi: 10.1371/journal.pone.0314748 (PMC11654971; doi:10.1371/journal.pone.0314748)
Supplement: S1 File — (DOCX) [file pone.0314748.s001.docx]

| SPONSOR | CHRU DE BREST |
| --- | --- |
| **CLINICAL TRIAL PROTOCOL SPICES** | |
| ACRONYM | SPICES_Phase 2 |
| TITLE | Scaling-up Packages of Interventions for Cardiovascular disease prevention in selected sites in Europe and Sub-Saharan Africa SPICES Study implementation phase  Setting up innovative interventions for the prevention of cardiovascular disease in Europe and Sub-Saharan Africa: SPICES Project _ Implementation phase |
| INDICATION(S) (TARGET) | Patients with cardiovascular risk factors |
| COORDINATING INVESTIGATOR | Pr Jean Yves Le Reste, EA SPURBO, Department of general practice, 22 avenue Camille Desmoulins, F-29200 Brest, France  lereste@univ-brest.fr |
| ASSOCIATED COORDINATING INVESTIGATORS | Dr Odorico Michele,  Dr Le Goff Delphine  EA SPURBO, Department of general practice, 22 avenue Camille Desmoulins, F-29200 Brest, France |
|  |  |
| PROTOCOL VERSION | V4.0 |
| PROTOCOL DATE | 05^th^ February 2021 |
| CPP | Approved on 09^th^ April 2019  By ethics committee (Comité de Protection des Personnes Sud Est IV, Lyon, France) |

SUBSTANTIAL MODIFICATIONS

| **protocol Version**  **(after modification)** | **Date** | **RATIONALE FOR AMENDMENT** |
| --- | --- | --- |
| **Version 2.0** | **24/09/2019** | **Modification of the justification for the number of participants to be included in the intervention phase** |
| **VERSION 3.0** | **07/12/2020** | **CHANGING THE NUMBER OF VISITS** |
| **VERSION 4.0** | **05/02/2021** | **CHANGES TO THE CONDUCT OF THE STUDY FOR PEOPLE RANDOMISED TO THE INTERVENTION GROUP** |

**Contents**

[1. General information 11](#_Toc138932518)

[1.1. Title 11](#_Toc138932519)

[1.2. Study sponsor 11](#_Toc138932520)

[1.2.1. Identity 11](#_Toc138932521)

[1.3. Investigators 11](#_Toc138932522)

[1.3.1. Coordinating investigator 11](#_Toc138932523)

[1.3.2. Associated investigators 11](#_Toc138932524)

[1.3.3. Associated scientists 11](#_Toc138932525)

[1.3.4. Methodologist - biostatistician 11](#_Toc138932526)

[1.3.5. Steering Committee 12](#_Toc138932527)

[2. Introduction and rationale 13](#_Toc138932528)

[2.1. Introduction and bacground 13](#_Toc138932529)

[2.2. Rationale for the study 14](#_Toc138932530)

[2.3.1. Benefits 15](#_Toc138932531)

[2.3.1.1. Individual profit 15](#_Toc138932532)

[2.3.1.2. Collective benefit 15](#_Toc138932533)

[2.3.2. Risks 15](#_Toc138932534)

[2.3.2.1. Individual risk 15](#_Toc138932535)

[2.3.2.2. Collective risk 15](#_Toc138932536)

[2.3.3. Benefit/risk balance 15](#_Toc138932537)

[2.4. Declaration that the research will be conducted in accordance with the protocol, good clinical practice and the legislative and current regulatory provisions 15](#_Toc138932538)

[3. Objectives 16](#_Toc138932539)

[3.1. Main objective 16](#_Toc138932540)

[3.2. Secondary objectives 16](#_Toc138932541)

[4. Study design and outcomes 16](#_Toc138932542)

[4.1. Qualification of research and investigators 16](#_Toc138932543)

[4.2. Primary endpoint 16](#_Toc138932544)

[4.3. Secondary end points 17](#_Toc138932545)

[5. Study population 17](#_Toc138932546)

[5.1. Inclusion criteria 17](#_Toc138932547)

[5.2. Non-inclusion criteria 17](#_Toc138932548)

[5.3. Population description 18](#_Toc138932549)

[6. Study description 18](#_Toc138932550)

[6.1. Inclusion 18](#_Toc138932551)

[6.2. Follow-up of people screened for moderate cardiovascular risk 19](#_Toc138932552)

[6.3. Study process 21](#_Toc138932553)

[6.4. Identifying the data collected in the observation notebook 22](#_Toc138932554)

[6.5. Expected duration of participation and description of duration of all trial periods, including follow-up, if applicable 23](#_Toc138932555)

[6.6. Support for research 23](#_Toc138932556)

[6.7. Withdrawals 23](#_Toc138932557)

[7. Safety assessment 23](#_Toc138932558)

[8. Statistics 23](#_Toc138932559)

[8.1. Description of planned statistical methods, 24](#_Toc138932560)

[8.2. Expected number of people to be included in the research, and expected number of people in each research location with statistical justification 24](#_Toc138932561)

[8.3. Method for taking into account missing, unused or invalid data 24](#_Toc138932562)

[9. Right of access to data 24](#_Toc138932563)

[9.1. Access to data 24](#_Toc138932564)

[9.2. Source documents 25](#_Toc138932565)

[9.3. Data confidentiality 25](#_Toc138932566)

[10. Quality control and assurance 25](#_Toc138932567)

[11. Ethical considerations 26](#_Toc138932568)

[11.1. Individual Protection Committee 26](#_Toc138932569)

[11.2. Substantial modification 26](#_Toc138932570)

[11.3. Participant information and consent 26](#_Toc138932571)

[12.1. Observation book electronic 26](#_Toc138932572)

[12.2. Data input and output 27](#_Toc138932573)

[12.3. Data protection authority (CNIL) 27](#_Toc138932574)

[12.4. Archiving 27](#_Toc138932575)

[13. Insurance 27](#_Toc138932576)

[14. Feasibility 27](#_Toc138932577)

[15. Final report and publishing rules 28](#_Toc138932578)

[16. References 28](#_Toc138932579)

**Summary**

| Title | Setting up innovative interventions for the prevention of cardiovascular disease in Europe and Sub-Saharan Africa - SPICES project Implementation phase |
| --- | --- |
| sponsor | Brest University Hospital  2 Avenue Foch  29609 Brest Cedex |
| Investigator Coordinator | Professor Jean Yves Le Reste  EA SPURBO, DUMG Brest. 22 avenue Camille Desmoulins, 29200 BREST CEDEX |
| Associate coordinating investigators | Dr Odorico Michele,  Dr Le Goff Delphine  EA SPURBO, DUMG Brest. 22 avenue Camille Desmoulins, 29200 BREST CEDEX |
| Version of protocol | Version 4.0 of 05/02/2021 |
| Background/ scientific rational | Cardiovascular disease (CVD) is the leading cause of death worldwide. 17.5 million people died from CVD in 2012 (31% of all causes of death). (1). In Europe, more than 50% of deaths are due to CVD (2). CVD mortality rates are highest in the lowest socio-economic groups. Three quarters of CVD deaths occur in developing countries (3). By 2030, it is estimated that CVD will be responsible for more deaths than the sum of infectious, nutritional, maternal and perinatal diseases in developing countries (4). The lack of an adequate primary care network in developing countries limits the detection and treatment of people with CVD. As a result, these people do not benefit from appropriate prevention, are diagnosed late and remain disabled or die at an early age, resulting in significant additional costs for families and at macroeconomic level (5).  Measures to prevent cardiovascular disease risk factors (CVDR) have been shown to be effective (6). Large-scale interventions are possible (policies against smoking and unfavourable eating habits, encouraging physical activity, etc.). Action is also possible on an individual level, both in primary prevention (combating CVDR) and secondary prevention, where numerous treatments have been shown to be effective (6). These interventions are effective and cost-effective from a macroeconomic point of view. It has been estimated that the cost of such interventions would not exceed 4% of healthcare expenditure in developing countries and 1-2% in developped countries (7).  The World Health Organisation (WHO) stresses the importance of the triad of patient and family, community and health professionals. Results are only possible when these three components work together towards the same goal (8). Numerous studies show the benefits of involving people in their own care, in both developped and developing countries (9) (10).  The SPICES project is inspired by the progress made in the treatment of HIV/AIDS in Sub-Saharan Africa (SSA) and in the management of chronic diseases thanks to the ICCC Framework (Innovative Care for Chronic Conditions), a WHO plan (8). As far as HIV treatment is concerned, these interventions have demonstrated their efficacy and cost-effectiveness in many SSA countries (11) (12) (13) (14) both in terms of disease control and compliance (15) (16). These data on communicable and infectious diseases seem to be transposable to non-communicable diseases.  These projects were born out of the realisation that the care model used in developped countries (individual patient approach, centred on the hospital and the specialist, with regular clinical and paraclinical follow-up) could not be transposed to developing countries, due to limited human, technical and financial resources. It was also becoming increasingly difficult to maintain this model in developed countries, where resources are becoming limited. New approaches need to be developed to make healthcare systems more efficient.  A paradigm shift is needed to improve CVD control in a more cost-effective way.  The SPICES project integrates the most up-to-date knowledge to improve the prevention and control of CVD in high-, middle- and low-income countries.  Both developped and developing countries are involved in the study. The sites selected are France, the United Kingdom, Belgium, South Africa and Uganda.  Some of the main thrusts of the ICCC Framework will be developed in SPICES :  - Improving the efficiency of healthcare professionals through the delegation of skills and appropriate training,  - centring care around the patient and their family and, more generally, their carers,  - simplify monitoring and treatment protocols,  - Supporting patients in their communities, with an emphasis on prevention, information and patient education.  An initial observation phase in the various countries enabled us to take stock of the situation and target the most appropriate interventions.  The next steps are to implement these interventions (delegation of skills, information and screening campaigns, improving the availability of treatments, measures to improve compliance, etc.) and to evaluate them.  This study, carried out in France and integrated into the SPICES project, will test the best non-pharmacological interventions selected in the community and by the community. |
| Main objective | To evaluate the effectiveness of a community-led behaviour change programme and brief intervention for people at moderate cardiovascular risk (according to the Interheart risk score) in reducing cardiovascular risk versus brief intervention. |
| Secondary Objectives | Identify and assess the obstacles and barriers to carrying out the intervention:   - During the screening phase - During the intervention phase - For those involved in screening and intervention - With participants |
| Primary endpoint | Comparison of the mean Non Laboratory Interheart risk score between the two arms at 24 months. |
| Secondary End Points | Secondary endpoints at 24 months:   - Quality of life assessed by the WHOQOL brief scale, - BMI reduction, - Level of smoking (Reduction in the number of smokers/reduction in the number of cigarettes per smoker) - Improving nutrition using the DASH Q, - Level of physical activity using the IPAQ questionnaire, - Alcohol consumption by collecting declared weekly alcohol consumption.   Tertiary evaluation criteria :   - At 12 months : - Non-Laboratory Interheart risk score average - At 6, 12 and 18 months: - Quality of life assessed by the WHOQOL brief scale, - BMI reduction, - Level of smoking (Reduction in the number of smokers/reduction in the number of cigarettes per smoker) - Improving nutrition using the DASH Q, - Level of physical activity using the IPAQ questionnaire, - Alcohol consumption by collecting declared weekly alcohol consumption   Implementation assessment criteria at 6, 12, 18 and 24 months:   - Number of people screened, number categorised as green, orange and red. - Number of people joining the SPICES programme (counted at the first SPICES meeting) - Number of people carrying out the entire SPICES intervention (75% of all meetings) - Cost of intervention - Barriers and facilitators to intervention   Qualitative assessment of screeners, intervention and resource persons |
| Methodology /study design | Randomised controlled trial |
| inclusion criteria | One group per phase :  - Screening phase :  From the general population, all people aged over 18 living or working in the Centre Bretagne region (including the Centre Ouest Bretagne, Pleyben and Callac regions).  - Randomised control trial (RCT) :  All people screened with a moderate cardiovascular risk score according to the Interheart clinical risk score (score of 9 to 15) in the screening phase. |
| Non-inclusion criteria | -For participants of the screening phase :  Pregnancy  Age under 18  Patients undergoing secondary cardiovascular prevention  People who do not live or work in Centre Bretagne (Pays Centre Ouest Bretagne and Pays de Pleyben et Callac)  For participants of the RCT, a low (<9) or high (>15) cardiovascular risk score according to the Interheart score. |
| Strategies/procedures | 1,000 people with a Non Laboratory Interheart risk score of 9 to 15 (moderate risk) will be included in the study in around 70 municipalities.    Around 250 screeners will be trained to use the Non Laboratory Interheart risk score and to include people at moderate cardiovascular risk (CVDR) in SPICES. These screeners will be recruited from the general population, from people with an interest in health promotion. Among these 250 screeners, 40 trainers will be trained in behaviour change techniques during a two-day session (run in France by UNAFORMEC, a certified training association). These trainers will be named community champions. A one-hour refresher course will follow this training every 3 months for the duration of the intervention.  The screeners and community champions will be supported during the intervention by family medecine interns under the supervision of the research team.  People with a high CVDR will receive brief advice and will be advised to consult their referring doctor.  People with a low CVDR will receive positive reinforcement. People with a moderate CVDR will be randomised to the intervention or control group.  People with moderate CVDR in the control group will receive brief advice regarding their Interheart score, while people with moderate CVDR in the intervention group will receive brief advice followed by a multi-behavioural intervention adapted to local resources. Increased physical activity will be recommended as a combination of aerobic and resistance activity. Diet will follow the Dash Diet recommendations. Tobacco and alcohol reduction will be proposed. The intervention groups will hold an initial action research meeting between people with moderate CVDR and community leaders. The aim of this meeting will be to create a map of external resources in the area supporting the improvement of modifiable risk factors (sports associations, for example). The groups will then meet regularly, face-to-face and/or remotely, for 2 years under the aegis of the trainer to work on modifying the risk factors of each individual. |
| Number of participants | 1000 participants |
| Number of centers | 2 centers  As this is a general population study, it was decided that all patients would be recruited under the aegis of 3 principal investigators from 2 centres (the Lanmeur and La Forest Landerneau centres, the practices of the coordinating investigator and the associate investigators respectively). |
| duration of research | Length of inclusion period: 12 months  Duration of participation for each patient: two years  Total duration of the study: 3 years |
| study impact | To validate an effective model of non-drug primary cardiovascular prevention engaging community champions and citizens. |

**Study glossary**

| ANSM | French National Agency for the Safety of Medicines and Health Products |
| --- | --- |
| AMM | Marketing Authorisation |
| ARC | Clinical Research Associate |
| ART | Anti Retroviral Therapy |
| ASS | Sub-Saharan Africa |
| BPC | Good Clinical Practice |
| CHRU | University Regional Hospital |
| CI | Ischaemic heart disease |
| PPC | Individual Protection Committee |
| CNIL | Commission Nationale de l'Informatique et des Libertés (French Data Protection Authority) |
| COB | Central West Brittany |
| EvIG | Serious Adverse Event |
| EIG | Serious Adverse Effect |
| ICH | International Conference on Harmonization |
| BMI | Body Mass Index |
| IPAQ Short | International Physical Activity Questionnaire |
| MCV | Cardiovascular disease |
| WHO | World Health Organisation |
| PVD | Developing countries |
| CVDR | Cardiovascular disease risk factors |
| SUSAR | Suspected Unexpected Serious Adverse Reaction |
| WHO Qol brief | World Health Organization Quality of Life scale |

1. General information
   1. Title

**Setting up innovative interventions for the prevention of cardiovascular disease in Europe and Sub-Saharan Africa - SPICES project Implementation phase**

- 1. Study sponsor
     1. Identity

Brest University Hospital

2 avenue Foch

29609 Brest Cedex

- - 1. Study coordination

Délégation à la Recherche Clinique et à l’Innovation

CHRU de Brest

2 Avenue Foch

29609 Brest Cedex

France

- 1. Investigators
     1. Coordinating investigator

Professor Jean Yves Le Reste

EA 7479 SPURBO, DUMG Brest. 22 avenue Camille Desmoulins, 29200 BREST CEDEX

Tel : 02 98 67 51 03

Email : [lereste@univ-brest.fr](mailto:lereste@univ-brest.fr)

- - 1. Associated investigators

Dr Odorico Michele - Tel : 02 98 67 51 03 - Email : [m.odoricobrest@gmail.com](mailto:m.odoricobrest@gmail.com)

Dr Le Goff Delphine - Tel : 02 98 67 51 03 - Email : [docteurdlegoff@gmail.com](file:///C:\chu-brest\Public\RECHERCHE%20BIOMEDICALE\Privé\C:\Users\Delphine%20Le%20Goff\AppData\Roaming\Microsoft\Word\docteurdlegoff@gmail.com)

EA 7479 SPURBO, DUMG Brest. 22 avenue Camille Desmoulins, 29200 BREST CEDEX

- - 1. Associated scientists

Dr Linda Gibson, Prof./Dr. Harm van Marwijk, Prof./Dr. Jean Yves LE RESTE, Dr. Geofrey Musinguzi, Prof./Dr. Paul Van Royen, Prof./Dr. Hilde Bastiaens, Prof Tholene Sodi.

- - 1. Methodologist - biostatistician

Professor Jean-Yves Le Reste, EA 7479 SPURBO, DUMG Brest. 22 avenue Camille Desmoulins, 29200 BREST CEDEX

Tel: 02 98 67 51 03 / Email: [lereste@univ-brest.fr](mailto:lereste@univ-brest.fr)

Associate professor Antoine Dany, EA 7479 SPURBO, DUMG Brest. 22 avenue Camille Desmoulins, 29200 BREST CEDEX/ Email : [antoine.dany@univ-brest.fr](mailto:antoine.dany@univ-brest.fr)

Data Management Unit (UGD), DRCI - Hôpital La Cavale Blanche - Boulevard Tanguy Prigent - 29609 Brest Cedex. (Head Emmanuel Nowak)

- - 1. Steering Committee

Professor Jean-Yves Le Reste, EA 7479 SPURBO, DUMG Brest. 22 avenue Camille Desmoulins, 29200 BREST CEDEX

Tel: 02 98 67 51 03 / Email: [lereste@univ-brest.fr](mailto:lereste@univ-brest.fr)

Dr Le Goff Delphine - EA 7479 SPURBO, DUMG Brest. 22 avenue Camille Desmoulins, 29200 BREST CEDEX Tel : 02 98 67 51 03 - Email : [docteurdlegoff@gmail.com](file:///C:\chu-brest\Public\RECHERCHE%20BIOMEDICALE\Privé\C:\Users\Delphine%20Le%20Goff\AppData\Roaming\Microsoft\Word\docteurdlegoff@gmail.com)

Dr Odorico Michele - EA 7479 SPURBO, DUMG Brest. 22 avenue Camille Desmoulins, 29200 BREST CEDEX Tel : 02 98 67 51 03 - Email : [m.odoricobrest@gmail.com](mailto:m.odoricobrest@gmail.com)

Dr Dany Antoine - EA 7479 SPURBO, DUMG Brest. 22 avenue Camille Desmoulins, 29200 BREST CEDEX Tel : 02 98 67 51 03 - Email : [antoine.dany@univ-brest.fr](mailto:antoine.dany@univ-brest.fr)

Prof./Dr. Hilde Bastiaens, Researcher Primary and Interdisciplinary Care, Universiteit Antwerpen, Antwerpen, Belgium, [hilde.bastiens@uantwerpen.be](mailto:hilde.bastiens@uantwerpen.be)

Sybil Anthierens, Primary and Interdisciplinary Care, Universiteit Antwerpen, Antwerp Belgium, [Sibyl.Anthierens@uantwerpen.be](mailto:Sibyl.Anthierens@uantwerpen.be)

Naomi Aerts, Primary and Interdisciplinary Care, Universiteit Antwerpen, Antwerp Belgium, [naomi.aerts@uantwerpen.be](mailto:naomi.aerts@uantwerpen.be)

Prof./Dr. Paul Van Royen, Primary and Interdisciplinary Care, Universiteit Antwerpen, Antwerp Belgium

Dr Linda Gibson, Senior Lecturer, School of Social Sciences Goldsmith Nottingham,

[linda.gibson@ntu.ac.uk](mailto:linda.gibson@ntu.ac.uk)

Mr Mark Bowyer SPICES Project Coordinator, Research Fellow in Public Health, Room 432 Chaucer Building School of Social Sciences Nottingham Trent University 0115 8485574 [mark.bowyer@ntu.ac.uk](mailto:mark.bowyer@ntu.ac.uk)

Prof./Dr. Harm van Marwijk, Professor in General Practice, Mayfield House Room 318a, University of Brighton, Falmer, Brighton BN1 9PH E: H.VanMarwijk@bsms.ac.ukT(direct line): 01273 644774 T (mobile): 07481527239

Papreen Nahar, University of Brighton, Falmer, Brighton BN1 9PH [P.Nahar@bsms.ac.uk](mailto:P.Nahar@bsms.ac.uk)

Dr. Geofrey Musinguzi Research Fellow Disease Control and Environmental Health Makerere University School of Public Health Kampala Uganda, [mgeof@musph.ac.ug](mailto:mgeof@musph.ac.ug)

Prof Tholene Sodi. Psychology Polokwane South Africa, [Tholene.Sodi@ul.ac.za](mailto:Tholene.Sodi@ul.ac.za)

Prof Mbuyiselo Douglas, SPICES Project Coordinator, Department of Psychology T 015 28 3505, [mbuyiselo.douglas@ul.ca.za](mailto:mbuyiselo.douglas@ul.ca.za)

1. Introduction and rationale
   1. Introduction and bacground

Cardiovascular diseases (CVD) are the leading cause of death worldwide. 17.5 million people died from CVD in 2012 (31% of all causes of death). (1). In Europe, more than 50% of deaths are due to CVD (2).

Measures to prevent cardiovascular risk factors (CVDRs) have been shown to be effective. In the general population, a 1% reduction in cholesterol could reduce mortality from ischaemic heart disease (IHD) by 2 to 4%; a 1% reduction in the prevalence of smoking would reduce numbers of deaths per year from IHD; a 1% reduction in diastolic blood pressure could prevent numerous deaths from IHD (6).

CVD mortality rates are higher in lower socio-economic groups. Three quarters of CVD deaths occur in developing countries (3). It is estimated that by 2030, CVD will be responsible for more deaths than the sum of infectious, nutritional, maternal and perinatal diseases in developing countries (4).

Improvements in the management of CVD are unevenly distributed, with disadvantaged groups benefiting the least of prevention and treatments (6).

The lack of an adequate primary care network in developing countries limits the detection and treatment of patients with cardiovascular risk factors (CVDR). As a result, these patients do not benefit from appropriate prevention, are diagnosed late and remain disabled or die at an early age, resulting in significant additional costs for families and at macroeconomic level (6).

Interventions are possible on a large scale (policies against smoking and unhealthy eating habits, encouraging transport by bicycle, etc.) but also on an individual level, both in primary prevention (combating CVDR) and secondary prevention, where numerous treatments have proven their effectiveness.

These types of intervention are effective and also cost-effective from a macro-economic point of view. It has been estimated that the cost of such interventions would not exceed 4% of healthcare expenditure in developing countries and 1-2% in developped countries (7).

The WHO (World Health Organisation) stresses the importance of the triad of patient and family, community and health professionals. Results are only possible when these three components work together towards the same objective (8). Involving patients and their families in decision-making and in planning the care pathway improves the effectiveness and cost-effectiveness of treatments (17).

Numerous studies show the importance of involving patients in their care, both in developped and developing countries (9) (10).

Studies also highlight the effectiveness of measures linked to new technologies (dedicated websites, SMS reminders, email, etc. (18)) as well as education, advice and information networks set up by and for patients, families and healthcare staff (10) (19).

The SPICES project was born out of the statement that a paradigm shift is needed to improve CVD control in a more cost-effective way.

- 1. Rationale for the study

The SPICES project is inspired by the progress made in the treatment of HIV/AIDS in Sub-Saharan Africa (SSA) and in the ICCC Framework (Innovative Care for Chronic Conditions), the WHO's plan for the management of chronic diseases (8).

These projects were prompted by the observation that the care model used in developped countries (individual approach to the patient, centred on the hospital and the specialist, with regular clinical and paraclinical follow-up) could not be transposed to developing countries, due to the limited human, technical and financial resources available. It was also becoming increasingly difficult to maintain this model in developed countries, where resources are becoming limited. New approaches need to be developed to make healthcare systems more efficient.

An innovative approach was needed to control chronic diseases.

As far as HIV treatment is concerned, the WHO has proposed a new plan with four main thrusts: simplification of monitoring and treatment protocols, facilitation of access to antiretroviral therapy (ART) via local dispensaries, delegation of tasks and involvement of the population and patients in the organisation of this new care system (20). Nurses, for example, have been trained in the implementation and monitoring of ART. Information campaigns have been conducted to improve compliance, and members of local communities have been trained to share information and provide individual support. These interventions have proven to be effective and cost-effective in many countries in SSA (11) (12) (13) (14) both in terms of disease control and compliance (15) (16). These data on communicable and infectious diseases seem to be transposable to non-communicable diseases.

Following this intervention, in 2002 the WHO presented the ICCC (Innovative Care for Chronic Conditions), a new plan to improve the management of chronic diseases.

This plan includes 8 main points: adopting a global approach to chronic diseases, adapting health policies, creating a care system integrated into the community, organising local policies, improving the effectiveness of health professionals, putting the patient and his or her family at the centre of the care pathway, supporting patients in their communities and focusing on prevention (8).

Some points of the ICCC project are developed in SPICES: improving the efficiency of healthcare professionals, centring care around the patient and his or her family, supporting patients in their community and insisting on prevention.

The interventions would target different levels.

An initial observation phase will enable the research team to identify the factors that limit and promote good CVD control and compliance in the various countries.

The following step will involve setting up or supporting existing networks in the various communities, and working with institutions to promote screening, education and information campaigns.

Training for healthcare professionals and citizens is considered, in particular training for community health workers in developing countries. This training will allow delegating the skills needed to set up screening and monitoring for CVDR. Some participants will be trained to play an advisory and support role for their peers (patient-expert model). The participant's formal or informal carers will also be fundamental in motivating them to follow the recommendations and follow the lifestyle modifications and treatments. Reminders using email and text messaging should also be explored.

The SPICES project aims to integrate current knowledge into new studies to improve the prevention and control of CVD in high-income countries (PRE: France, UK, Belgium), middle-income countries (PRI: South Africa) and low-income countries (PRF: Uganda).

This study, carried out in France and integrated into the SPICES project, will test the best non-pharmacological interventions selected in the community and by the community.

- 1. Benefit- Risk
     1. Benefits
        1. Individual profit

Participants can expect benefits in CVDR reduction. These benefits will be measured by changes in the Non Laboratory Interheart risk score, assessed by the research team.

- - - 1. Collective benefit

A collective benefit is expected on :

- Optimising the management of modifiable risk factors for cardiovascular disease
- Validation of a clinically effective and profitable prevention model
  - 1. Risks
       1. Individual risk

The risks to which the participant is exposed are, on one hand, the risk of over-diagnosis linked to the screening strategy using the Non Laboratory Interheart risk score and, on the other hand, the risk of over-treatment in people detected as having a high CVDR according to the Non Laboratory Interheart risk score.

Finally, there are psychological risks, mainly increased anxiety and reduced self-confidence. There are no painful or physically demanding examinations.

- - - 1. Collective risk

No collective risk is expected.

- - 1. Benefit/risk balance

In terms of the expected benefits and the risks to individuals, the benefit/risk balance is in favour of the intervention.

- 1. Declaration that the research will be conducted in accordance with the protocol, good clinical practice and the legislative and current regulatory provisions

The sponsor and the investigator also undertake that this research will be conducted :

- in accordance with the protocol,

- in accordance with current French and international good clinical practice,

- in accordance with the French and internationanl laws and regulations.

1. Objectives
   1. Main objective

To evaluate the effectiveness of a community-led behaviour change programme and brief intervention for people at moderate cardiovascular risk (according to the Non Laboratory Interheart risk score) on CVDR reduction versus brief intervention.

- 1. Secondary objectives

Identify and assess the obstacles and barriers for carrying out the intervention:

- During the screening phase
- During the intervention phase (RCT)
- For those involved in screening and intervention
- For participants

1. Study design and outcomes
   1. Qualification of research and investigators

This study is a prospective interventional study with minimal risks and constraints, not involving health products, according to the following points: "1. random allocation of procedure(s), or diagnostic or medical strategies or intervention(s) of current practice to a person or a group of persons" and "11. Interviews, observations and questionnaires, the results of which, in accordance with the protocol, may lead to changes in the participant's usual medical care and which therefore do not fall within the scope of the research mentioned in 3° of Article L.1121-1 of the Public Health Code" of the Order of 12 April 2018 establishing the list of research mentioned in 2° of Article L.1121-1 of the Public Health Code.

This study is a preventive study which forms part of the 6 fundamental skills of family practice*.*

The investigators are practitioners active in the field of primary care and networking (Professor JY Le Reste, head of the university department of family practice in Brest):

- Pr Le Reste heads an EA focusing on the study of Primary Care and Prevention in the general population (SPURBO) and has published a large amount of research on this subject.
- Dr Le Goff has led a number of behaviour change groups at the diabetes network in Orléans and is an expert in motivational interviewing training.

These investigators, qualified persons in accordance with article 1122-1 of the Public Health Code, will be represented by family practice interns who will collect consent and data. These interns will be qualified persons under article 1122-1 of the Public Health Code, as they will have received appropriate training in the research protocol, as well as training in Good Clinical Practice and the use of the observation notebook, all under the aegis of the doctor-investigators and the Clinical Research and Innovation Delegation, which is ISO 9001-2015 certified for its activities in promoting and coordinating academic interventional clinical trials.

- 1. Primary endpoint

Comparison of the mean Non Laboratory Interheart risk score between the two arms at 24 months.

- 1. Secondary end points

Those secondary clinical endpoints will be measured at 24 months:

- Quality of life assessed by the WHOQOL brief scale,
- BMI reduction,
- Level of smoking (Reduction in the number of smokers/reduction in the number of cigarettes per smoker)
- Improving nutrition using the DASH Q,
- Level of physical activity using the IPAQ questionnaire,
- Alcohol consumption by collecting declared weekly alcohol consumption.

Tertiary evaluation criteria :

- At 12 months :
- Non Laboratory Interheart risk score
- At 6, 12 and 18 months:
- Quality of life assessed by the WHOQOL brief scale,
- BMI reduction,
- Level of smoking (Reduction in the number of smokers/reduction in the number of cigarettes per smoker)
- Improving nutrition using the DASH Q,
- Level of physical activity using the IPAQ questionnaire,
- Alcohol consumption by collecting declared weekly alcohol consumption

Implementation assessment criteria at 6, 12, 18 and 24 months:

- Number of people screened, number categorised as green, orange and red.
- Number of people joining the SPICES programme (counted at the first SPICES meeting)
- Number of people carrying out the entire SPICES intervention (75% of all meetings)
- Cost of intervention
- Barriers and facilitators to intervention
- Qualitative assessment of screeners, intervention and resource persons

1. Study population
   1. Inclusion criteria

One group per phase :

- Screening phase :

From the general population, all people aged over 18 living or working in the Centre Bretagne region (including the Centre Ouest Bretagne, Pleyben and Callac regions).

- Randomised control trial (RCT) :

All people screened with a moderate cardiovascular risk score according to the Interheart clinical risk score (score of 9 to 15) in the screening phase.

- 1. Non-inclusion criteria

-For participants of the screening phase :

- Pregnancy
- Age under 18
- Patients undergoing secondary cardiovascular prevention
- People who do not live or work in Centre Bretagne (Pays Centre Ouest Bretagne and Pays de Pleyben et Callac)

For participants of the RCT, a low (<9) or high (>15) cardiovascular risk score according to the Interheart score.

- 1. Population description

People will be screened and recruited from the general population by trained members of the public with an interest in health promotion, named screeners.

The 250 screeners will be recruited by the research team using the network of the stakeholders of the Pays COB and the Brest medical school, particularly among the students who carry out their mandatory preventative health service (medical students, nursing students, physiotherapy students) and the associations located in the Pays COB. The screening sites will be defined the stakeholders of the Pays COB. Screenings will take place in places where people live in the community: markets, supermarkets, associations, school outings, etc.

1. Study description
   1. Inclusion

Approximately 1,000 people with an Interheart Score of 9 to 15 (moderate risk) will be included in the SPICES study in around 70 municipalities from December 2019 to December 2020.

There are two steps to this study:

- The screening step: carried out in places frequented by the general population (markets, supermarkets, associations, school outings, etc.) in COB countries, outside healthcare facilities.

- The intervention step: After screening, the research team will contact people with a moderate CVDR (Non Laboratory InterHeart risk score orange from 9 to 15 inclusive) to include them into the SPICES study and to randomise them. This randomisation will random participation in either the control group or the intervention group.

Screening will be carried out on an opportunistic basis in places frequented by the general population (markets, supermarkets, associations, school outings, etc.) in the COB region, away from healthcare facilities. When the screeners make contact, all the information will be given verbally, and participants will be given a general information leaflet or flyer. This leaflet will explain the reason for screening, what the research team will do with the data collected and the step following screening, i.e. a new contact for patients with a moderate CVDR to join the Spices study. This prospectus will also include the specific contact details of the research team for the project, dedicated email and telephone number.

The screener will use the tablet to record participant’s informed consent to participate to the screening using the non-laboratory version of the Interheart score.

The score comprises eight items, comprising seven questions and the calculation of the waist-to-hip ratio. Screeners will be equipped with a tape measure.

The data will be entered via the tablet, which will be used to calculate the Interheart score and define the tercile in which the participant’s risk score falls.

A brief advice linked to the participant's risk score will be automatically provided by the software.

The advice will be given immediately by the screener:

- For people whose score is in the lowest tercile (green score): no change in lifestyle and positive reinforcement,
- For people whose score is in the moderate tercile (orange score): advice on lifestyle changes and a proposal to join the Spices study (=intervention group).
- For people whose score is in the top tercile (red score): advice to change lifestyle habits and referral to the referring doctor.

The brief advice provided during screening will be standardised minimum advice in line with the latest recommendations and will be identical in the two steps. It will include the health messages disseminated to the general public by the national plans identified during the situational analysis carried out at the start of the project (Programme national de réduction du tabagisme (PNRT); Programme national nutrition santé (PNNS); Plan Santé sport bien-être (SSBE); Plan national santé environnement (PNSE); Plan de santé au travail; Plan Accident Vasculaire Cérébral (Plan AVC); Plan Obésité).

Example: *« You have declared that you are an active smoker. Quitting smoking will reduce your risk of cardiovascular disease. You have stated that you do not eat enough fruit and vegetables. Eating fruit and vegetables every day will reduce your risk of cardiovascular disease. »*

The screeners will be supported during the intervention by family medecine interns under the supervision of the research team. These family medecine interns at the Faculty of Brest will be working on this project as part of their mandatory master thesis.

- 1. Follow-up of people screened for moderate cardiovascular risk
     1. Control/Intervention groups

After screening, the research team will contact people with a moderate CVDR (Non Laboratory InterHeart risk score orange from 9 to 15 included) to include them into the SPICES study and to randomise them. The software will random participants in either the control group or the intervention group.

Regardless of randomisation, participants will be individually interviewed by a member of the research team, who will measure the clinical endpoints at time zero of the intervention, and then at 6, 12, 18 and 24 months.

These clinical criteria consist of :

- Quality of life assessed by the WHOQOL brief scale,
- Weight, height and BMI,
- Smoking levels (reduction in the number of smokers/reduction in the number of cigarettes per smoker)
- Nutrition using the DASH Q questionnaire,
- Level of physical activity using the IPAQ short questionnaire,
- Alcohol consumption by collecting declared weekly alcohol consumption

6.2.2. The Control group

In the control group, people with a moderate CVDR will receive brief advice according to their Interheart score.

The brief advice provided during screening will be standardised minimum advice in line with the latest recommendations and will be identical in the two steps. It will include the health messages disseminated to the general public by the national plans identified during the situational analysis carried out at the start of the project (Programme national de réduction du tabagisme (PNRT); Programme national nutrition santé (PNNS); Plan Santé sport bien-être (SSBE); Plan national santé environnement (PNSE); Plan de santé au travail; Plan Accident Vasculaire Cérébral (Plan AVC); Plan Obésité).

Example: *« You have declared that you are an active smoker. Quitting smoking will reduce your risk of cardiovascular disease. You have stated that you do not eat enough fruit and vegetables. Eating fruit and vegetables every day will reduce your risk of cardiovascular disease. »*

6.2.3. The Intervention group

6.2.3.1 Inclusion

In the intervention group, people with moderate CVDR will receive brief advice followed by a multi-behavioural intervention adapted to local resources.

The intervention was defined on the basis of a systematic review of international and national good practice recommendations for cardiovascular prevention.

The diet will follow the Dash Diet recommendations.

The reduction of tobacco and alcohol consumption will be proposed.

Increased physical activity will be recommended as a combination of aerobic and resistance activity.

There is no provision for compensation for people included in the intervention group. A review of the literature shows that financial incentives are not effective when used alone. On the other hand, when they are combined with a multimodal intervention, their effect is moderate and ceases when the funding is stopped (21). The aim of our project is to ensure that the intervention lasts beyond the end of the project. Secondly, financial incentives belong to a different category of the behaviour change taxonomy from that currently adopted by the SPICES consortium (including goal setting, problem solving and action planning) (22). This incentive would change the method of our intervention and create a divergence from the projects of the other members of the SPICES consortium.

6.2.3.1. Study follow-up for participants

In addition to contacts at 6, 12, 18 and 24 months, the psychological risks associated with the intervention will be collected with a specific question: "Did the participants experience any psychological effects associated with the research?

- 1. Study process

Each intervention group will hold an initial research action meeting between people with moderate CVDR and community leaders.

The aim of this meeting is to map external resources in the COB region that help the improvement of modifiable risk factors (sports associations, for example).

Each group will then meet regularly, face-to-face and/or remotely (by telephone or videoconference), under the aegis of the community champions, to work on the changes in each participant's risk factors. The one-hour sessions will be scheduled every fortnight for 2 months, then every month for 2 months, then every 3 months until the end of the study (13 sessions). These sessions will be behaviour change sessions, focusing on goal setting, action planning and problem solving. The community champions will lead these sessions and use motivational interviewing techniques.

Community champions will first take part in a 2-day training course with national experts (run in France by UNAFORMEC, a certified training association).

The community champions will be compensated for their time on a lump-sum basis. This is consistent with the interventions proposed by the other partners in the SPICES consortium.

At the same time as these sessions, the people in the intervention group will be contacted individually (face-to-face or remotely by telephone or videoconference) by the research team. The aim of this contact will be to help them make lifestyle changes to improve primary cardiovascular prevention.

The volunteer community champions will be supported by family medecine interns under the supervision of the research team. These family medecine interns of the Faculty of Brest will be working on this project as part of their mandatory master thesis. They will support the community champions, offering them logistical assistance (organising face-to-face or remote meetings), help in running their meetings and assisting the members of their groups to change their lifestyles in favour of better cardiovascular prevention.

At 6, 12, 18 and 24 months, various criteria will be measured by the research team:

- Quantitative implementation assessment criteria :
- Number of people screened, number categorised as green, orange and red.
- Number of people taking part in SPICES activities (the count will be made at the first SPICES meeting)
- Number of people carrying out the entire SPICES intervention (75% of all meetings)
- Cost of the intervention, taking into account the cost of initial training for screeners and community champions, equipment (tablets for screening and paper tools for community champions), group room hire costs, mileage allowance for community champions, an estimate of remuneration for community champions based on the minimum hourly wage and mileage allowance for participants in the intervention group.
- Qualitative implementation assessment criteria:
- The aim of collecting these criteria is to identify the barriers and facilitators to the intervention. It is based on a qualitative assessment of the screeners, the intervention and the resource people, in line with the recommendations of the Re-Aim model.
- Qualitative evaluation of screeners using focus-group methods and individual semi-structured interviews. A purposive sampling of screeners will be carried out. This will be based in particular on age, the number of people screened and the geographical location of the screening. Data collection will continue until the data is theoretically saturated. Data analysis will use a thematic analysis. Two researchers will analyse the data blindly and then collate the analyses.
- Qualitative evaluation of community champions using focus group methods and individual semi-structured interviews. A purposive sampling of community champions will be carried out. It will be based in particular on age and the geographical location of the sessions. Data collection will continue until the theoretical saturation point is reached. Data analysis will use a thematic analysis.. Two researchers will analyse the data blindly and then collate the analyses.
- Qualitative assessment of participants using focus group methods and individual semi-structured interviews. A purposive sampling of participants will be carried out. It will be based in particular on age and the geographical location of the sessions. Data collection will continue until the data is theoretically saturated. Data analysis will use a thematic analysis. Two researchers will analyse the data blindly and then collate the analyses.
- Qualitative assessment of non-participants using semi-structured individual interviews. Non-participants are defined as people who have been screened but have been lost to follow-up or have disappeared during the intervention. A purposive sampling of non-participants will be carried out. It will be based in particular on age and the geographical location of the sessions. Data collection will continue until the theoretical saturation point is reached. Data analysis will use a thematic analysis. Two researchers will analyse the data blindly and then collate the analyses.
  1. Identifying the data collected in the observation notebook

Data collected will include demographics, history, weight, height, BMI of participants, responses to Interheart questionnaires, Whoqol brief quality of life, IPAQ short physical activity, DASH-Q diet, self-reported level of smoking and self-reported level of alcohol consumption.

Demographics :

- Age
- Tobacco consumption according to Interheart (never smoked, former smoker, smoker of 1 to 5 cigarettes a day, 6 to 10 cigarettes a day, 11 to 15 cigarettes a day, 16 to 20 cigarettes a day, more than 20 cigarettes a day)
- Passive smoking, formulated according to Interheart
- Diet according to Interheart criteria (5 questions) and DASH-Q criteria (11 questions)
- Quality of life using the short Whoqol scale, 26 items exploring 4 areas: physical health, mental health, social relationships and the environment.
- Physical activity based on Interheart and IPAQ-short data (7 questions)

History

Family history: history of heart attack in fathers or mothers

Personal history: existence of type 2 diabetes, arterial hypertension, etc.

Clinical data :

- Weight, height and BMI of intervention participants
- Waist to hip ratio measured with a tape measure according to Interheart recommendations
- Professional and personal stress formulated according to Interheart
- Depression and related syndromes as formulated by Interheart
- Specific risk assessment question: did the participant experience any psychological effects related to the research?
  1. Expected duration of participation and description of duration of all trial periods, including follow-up, if applicable

The duration of screening and inclusion in the intervention and control groups will be one year.

The intervention will last two years.

The total duration of the intervention (screening + intervention) will be three years.

Participants will participate in the study for three years.

- 1. Support for research

There is no standard management of primary prevention patients in the community. The specific research management was created on the basis of the results of a structured review of the literature on the best current recommendations for good practice in primary care, adressing an adapted diet, adapted physical activity, reduction or cessation of smoking and alcohol, and weight loss.

- 1. Withdrawals
     1. Withdrawal of participants

Participants may withdraw their consent and ask to leave the study at any time and for any reason. In the event of premature withdrawal, the investigator must document the reasons as fully as possible.

The investigator may temporarily or permanently discontinue a participant’s participation in the study for any reason that is in the best interests of the participant, particularly in the case of serious adverse events.

If a participant is lost to follow-up, the investigator will make every effort to contact the person again*.*

- - 1. Total or partial arrest of the study

Unforeseen events or new information relating to the research, in the light of which the objectives of the study are unlikely to be achieved, may lead the sponsor to interrupt the study prematurely.

The CHRU de Brest reserves the right to discontinue the study at any time if the inclusion objectives are not met.

1. Safety assessment

As part of this study, investigators will report research-related adverse events occurring during the participation of recruited patients via the electronic CRF. The sponsor will receive an e-mail alert whenever an AE is entered into this CRF. This information will be used to create a register which will be used to draw up the final study report.

In accordance with the Public Health Code, new facts (defined in 12° of article R1123-46) will be reported to the CPP.

1. Statistics

Responsible for analysis: UGD - Brest Clinical Research and Innovation Delegation - CHRU de Brest - 2, avenue Foch - 29609 BREST cedex

All analyses were carried out using SAS and R software.

Analyses will be carried out with an alpha risk of 0.05

- 1. Description of planned statistical methods,

The general principles of the analysis are :

- The analysis will be conducted on "intention to treat".
- There is no intermediate analysis because there is no biomedical risk associated with the research.
  - Hierarchical analysis in two steps: 1) comparison of the mean Interheart between the two arms at a risk of alpha=0.05, then in the event of a statistically significant result 2) comparison of the 6 secondary endpoints between the two arms, using the Holm-Bonferroni correction to take account of the multiple comparisons carried out in this step. In accordance with the principles of hierarchical analysis, the tests in step 2) can only be performed demonstratively if the test in step 1) is statistically significant (p<0.05).
- Quantitative endpoints will be compared between the two arms using a Student's t test. Qualitative endpoints will be compared between the two groups using a Chi-2 test. Multivariate analyses will be performed secondarily using a linear model to adjust for potential confounding factors.
  1. Expected number of people to be included in the research, and expected number of people in each research location with statistical justification

The calculation of the number of participants is based on the comparison of the mean Interheart between the two arms: for a power of 80%, a risk alpha=0.05 and an effect size (mean difference / standard deviation) of 0.20 the number of participants required is 394 per arm. Including approximately 20% lost to follow-up, a total of 985 participants should be included. With a number of participants of 394 patients per arm and the assumption of an effect size of 0.20 for each secondary endpoint, the test power is approximately 68% for each secondary endpoint using the Holm-Bonferroni correction to account for multiple comparisons.

- 1. Method for taking into account missing, unused or invalid data

Missing data will be identified via the electronic CRF, and the investigators will be called back to complete the data. Incomplete files will be declared lost to follow-up.

1. Right of access to data
   1. Access to data

In accordance with GCP :

- the sponsor is responsible for obtaining the agreement of all parties involved in the research in order to guarantee direct access to all research sites, source data, source documents and reports for the purposes of quality control and audit by the sponsor,

- the investigators will make available to the persons responsible for monitoring, quality control or auditing the research, the documents and individual data strictly necessary for this control, in accordance with the legislative and regulatory provisions in force (articles L.1121-3 and R.5121-13 of the Public Health Code).

- 1. Source documents

Source documents, which are defined as any original document or object making it possible to prove the existence or accuracy of data or facts recorded during the [clinical study](http://134.157.220.13/urcest/sous_cadre.php?fich=Lexique/E.htm#essaiclinique), will be kept for 15 years by the investigator or by the hospital in the case of hospital medical records.

- 1. Data confidentiality

In accordance with the legal provisions adressing data confidentiality for persons responsible for research quality control (article L.1121-3 of the Public Health Code), in accordance with the legal provisions relating to the confidentiality of information adressing, especially, the nature of the products, the trials, the persons taking part in them and the results obtained (article R. 5121-13 of the Public Health Code), persons with direct access will take all necessary precautions to ensure the confidentiality of information relating to the trials, the persons taking part in them and, in particular, their identity and the results obtained.

These people, in the same way as the investigators themselves, are subject to professional secret (under the conditions defined by articles 226-13 and 226-14 of the French Penal Code).

During the research or at its conclusion, the data collected on the participants and transmitted to the sponsor by the investigators (or any other specialist) will be rendered anonymous.

Under no circumstances may the names or addresses of the persons concerned appear.

Only the first letter of the participant's surname and first name will be recorded, together with a coded number specific to the study indicating the order of inclusion of participants.

The sponsor will ensure that each person taking part in the research has given their written consent for access to individual data concerning them and strictly necessary for the quality control of the research.

1. Quality control and assurance

A Clinical Research Assistant (CRA) may be appointed by the sponsor. He or she will ensure that the study is carried out properly, that the data generated are collected in writing, documented, recorded and reported, in accordance with the Standard Operating Procedures implemented at the CHRU de Brest and in compliance with Good Clinical Practice and the current legislative and regulatory provisions.

The investigator and the members of his team agree to be available for Quality Control visits carried out at regular intervals by the CRA. During these visits, the following elements may be reviewed in accordance with the monitoring grade defined by the sponsor for the study:

- informed consent
- compliance with the study protocol and the procedures defined therein
- quality of the data collected in the observation book: accuracy, missing data, consistency of data with "source" documents (medical records, appointment books, original laboratory results, etc.)
- completion of questionnaires or scales

The investigators also agree to accept quality assurance audits by the sponsor and inspections by the competent authorities. All data, documents and reports may be the subject of audits and regulatory inspections, without the right to invoke medical confidentiality.

1. Ethical considerations
   1. Individual Protection Committee

The study protocol, information form and consent form will be submitted to the Comité de Protection des Personnes for approval. Notification of the CPP's favourable opinion will be sent to the study sponsor and the competent authority.

The trial will be performed in accordance with the recommendations guiding physicians in biomedical research involving human subjects adopted by the 18th World Medical Assembly (Helsinki, Finland, 1964 and later revisions), the Tri-Council Policy Statement and the ICH Guidelines.

- 1. Substantial modification

Any substantial modification made to the protocol by the investigator must be approved by the sponsor. Prior to implementation, the sponsor must obtain a favourable opinion from the CPP. If necessary, new consent will be obtained from the persons taking part in the research.

- 1. Participant information and consent

The investigator will inform each participant prior to inclusion in the trial, full and adequate verbal and written information regarding the objective and procedures of the trial and the possible risks involved, the necessary surveillance and safety measures, their right to refuse to participate in the study or the possibility of withdrawing at any time.

For people screened (the general population of the COB area), this information will be given orally and a general information letter such as a leaflet or flyer will be sent to them.

For people recruited with a moderate CVDR, all the information is given on an information and consent form given to the participant. The participant's free, informed and written consent will be obtained before definitive inclusion in the study by the trainer who will represent one of the 3 doctors declared as investigators. These community champions will all have received appropriate training, validated by a certificate giving them the authority of the declared investigators to present and sign the consent form.

A copy of the information and consent form signed by both parties will be given to the participant, and the trainer will keep a copy.

As this study is taking place in the general population, all the participants recruited will depend on the 3 declared investigators.

1. **Data recording and processing**
   1. Observation book electronic

All the information required by the protocol must be recorded in the observation books. Data should be collected as it is obtained and recorded explicitly in the notebooks. Any missing data should be coded.

This electronic observation book will be accessible via an Internet data collection medium provided to community champions.

If the research team fills in the case report form via the Internet, the CRA can view the data quickly and remotely. The investigator is responsible for the accuracy, quality and relevance of all data entered. In addition, when data is entered, it is immediately checked for consistency. As such, he must validate any change in value in the CRF. These changes are subject to an audit trail. A justification may be included as a comment.

A pdf copy of the observation notebooks signed by the investigator will be saved and given to the investigator for archiving.

- 1. Data input and output

Data will be entered electronically via the web browser RedCap.

- 1. Data protection authority (CNIL)

As this study requires the community champions to contact patients with moderate cardiovascular risk at the end of the screening period, it is necessary for the research team to have the full identity of the participants, as well as their telephone and postal contact details. This identifying data, which is strictly necessary for carrying out the research, will be kept only until the end of the study and will be accessible only by the research staff. The "Reference Methodology" (MR-001 - N°1749494v0 dated 11/03/2014) in application of the provisions of article 54 paragraph 5 of law n°78-17 of 6 January 1978 as amended therefore applies to this project.

- 1. Archiving

All study data will be stored in the investigating centers and on the sponsor's premises in accordance with the current regulations.

1. Insurance

The Sponsor will take out insurance covering its own civil liability and that of any doctor involved in carrying out the study, for the entire duration of the study. It will also provide full compensation for the harmful consequences of the research for the person who takes part in it and their dependants, unless it can prove that the damage is not attributable to its fault or to that of any other party involved, without any claim being made against it on the grounds of a third party or the voluntary withdrawal of the person who initially agreed to take part in the research.

1. Feasibility

In 2006, the Center Brittany region had a population of 103,674 people. There is an excess of cardiovascular deaths in the region, with a specific mortality index of 113 CVD-related deaths in the region compared to 100 in the general French population. The Interheart cardiovascular risk is divided into terciles for equal populations, which suggests that one-third of people screened have a moderate or orange CVDR. A total of 7,350 people would have to be screened to reach the number of participants to be included of 2,400 in all the arms, an estimate that includes those lost to follow-up during the intervention. Based on other general population screenings, it can be estimated that 30% of general population contacts refuse to undergo screening or to be included in the study. This brings the total number of contacts per screener to 9555, assuming 250 screeners, i.e. an average of 36.75 contacts per screener. The capacity to recruit screeners is high because of the creation of the preventative health service for medical and paramedical students and the service is consistent with the SPICES project (90 medical students in Brest, 90 nursing students in Pontivy, 30 physiotherapy students in Brest). Other training courses, such as preparatory classes in nursing, have shown an interest in the project because of a similar fit (40 students in a preparatory class in Gourin). Presentations to associations in the Centre Bretagne region offer further opportunities.

40 community champions are needed to perform the behavioral change sessions. Some fifteen contacts from associations in the area have shown an interest in this role, as have students from the citizen's prevention university. The research team benefits from the social expertise of the chair of the region's health commission and a regional coordinator, as well as the support of the COB regional council. The research team has expertise in behaviour change and communication techniques (training leading to qualifications in therapeutic education).

1. Final report and publishing rules

All actors who have contributed substantially in study design, data collection, data analysis and interpretation, manuscript preparation and critical revising, and in final manuscript version approval, will be affiliated as authors. The sponsor and funders will be acknowledged in the published manuscript.

A summary of the final report made in accordance with the reference plan of the appropriate authority will be send to this authority as well as to the EC within a year after the end of the clinical research, i.e. the last visit means the last followed up participant. The final report of the research will be written in collaboration with the coordinator, the sponsor and the biostatistician of the research trial. A final version should be endorsed by the signature of each of the investigators and be addressed to the sponsor within a brief deadline after the actual end of the research trial.

Publishing rules will follow the International Committee of Medical Journal Editors (ICMJE) authorship requirements.

1. References

1 (WHO) WHO. A Global Brief on Hypertension: Silent Killer, Global Public Health Crisis. 2013.

2. Nichols M, Townsend N, Scarborough P, Rayner M. Cardiovascular disease in Europe 2014: epidemiological update. Eur Heart J. 2014 Nov;35(42):2929.

3. Yusuf S, Reddy S, Ôunpuu S, Anand S. Global Burden of Cardiovascular Diseases. Circulation. 2001 Nov;104(22):2746-53.

4. Mathers CD, Loncar D. Projections of Global Mortality and Burden of Disease from 2002 to 2030. PLOS Med. 2006 Nov;3(11):e442.

5. WHO. Cardiovascular diseases (CVDs) [Internet]. WHO. World Health Organization; 2017 [cited 2018 Mar 5]. Available from: http://www.who.int/cardiovascular_diseases/en/

6. WHO | Cardiovascular diseases (CVDs). WHO. World Health Organization; 2017;

7. World Health Organization. WHO, Global action plan for the prevention and control of non-communicable diseases, 2013-2020. Geneva. WHO; 2013.

8. WHO | Innovative Care for Chronic Conditions: Building Blocks for Action. WHO.

9. Axelsson JM, Hallager S, Barfod TS. Antiretroviral therapy adherence strategies used by patients of a large HIV clinic in Lesotho. J Heal Popul Nutr. 2015 Dec;33(1):10.

10. Feachem RGA, Sekhri NK, White KL. Getting more for their dollar: a comparison of the NHS with California's Kaiser Permanente. BMJ. 2002;324(7330):135–41.

11. Bemelmans M, Van Den Akker T, Ford N, Philips M, Zachariah R, Harries A, et al. Providing universal access to antiretroviral therapy in Thyolo, Malawi through task shifting and decentralization of HIV/AIDS care. Trop Med Int Heal. 2010 Dec;15(12):1413-20.

12. Boulle A, Cutsem G Van, Hilderbrand K, Cragg C, Abrahams M, Mathee S, et al. Seven-year experience of a primary care antiretroviral treatment programme in Khayelitsha, South Africa. Lippincott Williams & Wilkins. 2010;24:563-72.

13. Stringer JSA, Zulu I, Levy J, Stringer EM, Mwango A, Chi BH, et al. Rapid scale-up of antiretroviral therapy at primary care sites in Zambia: feasibility and early outcomes. JAMA. 2006 Aug;296(7):782-93.

14. Fox MP, Rosen S. Patient retention in antiretroviral therapy programmes up to three years on treatment in sub-Saharan Africa, 2007-2009: systematic review. Trop Med Int Heal. 2010 Jun;15:1-15.

15. Jaffar S, Amuron B, Foster S, Birungi J, Levin J, Namara G, et al. Rates of virological failure in patients treated in a home-based versus a facility-based HIV-care model in Jinja, southeast Uganda: a cluster-randomised equivalence trial. Lancet. 2009 Dec;374(9707):2080-9.

16. Zachariah R, Teck R, Buhendwa L, Fitzerland M, Labana S, Chinji C, et al. Community support is associated with better antiretroviral treatment outcomes in a resource-limited rural district in Malawi. Trans R Soc Trop Med Hyg. 2007 Jan;101(1):79-84.

17. Holman H, Lorig K. Patients as partners in managing chronic disease. BMJ Br Med J. 2000 Feb;320(7234):526-7.

18. Hüsler J, Peters T, bridges.org. Evaluation of the On Cue Compliance Service Pilot. Testing the use of SMS reminders in the treatment of Tuberculosis in Cape Town, South Africa. City of Cape Town Health Directorate and the International Development Research Council (IDRC);

19. Ellen N, Martin M. Caring For People With Chronic Conditions: A Health System Perspective: A Health System Perspective. McGraw-Hill Education (UK); 2008. 283 p.

20. Van Olmen J, Schellevis F, Van Damme W, Kegels G, Rasschaert F. Management of Chronic Diseases in Sub-Saharan Africa: Cross-Fertilisation between HIV/AIDS and Diabetes Care. J Trop Med. Hindawi; 2012 Nov;2012:1-10.
