# Supplementary material for: Innovative population-based strategies for primary prevention of cardiovascular disease: A 2-year randomised control trial evaluating behavioral change led by community champions versus brief advice
Source: PLoS One. 2024 Dec 18;19(12):e0314748. doi: 10.1371/journal.pone.0314748 (PMC11654971; doi:10.1371/journal.pone.0314748)
Supplement: S2 Appendix — (DOCX) [file pone.0314748.s004.docx]

**Appendix B: Analysis of secondary outcomes**

**WHOQOL**

I. Physical WHOQOL

| **Criteria** | **INTERVENTION Group**  **Mean (standard deviation)**  **(N=272)** | **CONTROL Group**  **Mean (standard deviation)**  **(N=264)** | ***Adjusted difference**  **INTERVENTION– CONTROL**  **Mean (95% CI)** |
| --- | --- | --- | --- |
| **M0** | 72.28 (15.19)  N =270 (2 missing) | 73.86 (15.47)  N= 264 (0 missing) |  |
| **M6** | 73.05 (13.78)  N= 181 (91 missing) | 72.97 (15.27)  N=208 (56 missing) |  |
| **Δ (M6-M0)** | 0.42 (11.16) | -1.15 (12.93) | 1.03 (-1.15; 3.21)  p= 0.36 |
| **M12** | 71.70 (16.19)  N= 159 (113 missing) | 71.58 (15.21)  N= 191 (73 missing) |  |
| **Δ (M12-M0)** | -0.26 (13.41) | -2.99 (12.80) | 1.83 (-0.74; 4.40)  p=0.16 |
| **M18** | 67.35 (16.63)  N= 126 (146 missing) | 70.34 (17.97)  N=157 (107 missing) |  |
| **Δ (M18-M0)** | -3.73 (12.92) | -3.69 (15.02) | -0.89 (-4.07; 2.28)  p= 0.58 |
| **M24** | 66.53 (17.26)  N= 110 (162 missing) | 70.55 (17.94)  N= 147 (117 missing) |  |
| **Δ (M24-M0)** | -4.08 (13.82) | -3.33 (14.55) | -1.68 (-5.06; 1.70)  p= 0.33 |

* Adjusted difference on the WHOQOL-PH score at M0 (the difference is in favour of the IG if it is positive).

II. Psychological WHOQOL

| **Criteria** | **INTERVENTION Group**  **Mean (standard deviation)**  **(N=272)** | **CONTROL Group**  **Mean (standard deviation)**  **(N=264)** | ***Adjusted difference**  **INTERVENTION– CONTROL**  **Mean (95% CI)** |
| --- | --- | --- | --- |
| **M0** | 66.03 (14.69)  N = 270 (2 missing) | 65.75 (15.92)  N= 264 (0 missing) |  |
| **M6** | 65.33 (15.56)  N=181 (91 missing) | 65.50 (15.49)  N=208 (56 missing) |  |
| **Δ (M6-M0)** | -0.67 (12.50) | -0.56 (10.91) | -0.14 (-2.32; 2.03)  p= 0.90 |
| **M12** | 65.93 (16.95)  N=159 (113 missing) | 66.86 (15.35)  N=191 (73 missing) |  |
| **Δ (M12-M0)** | 1.05 (14.05) | 0.92 (12.94) | -0.21 (-2.85; 2.43)  p= 0.88 |
| **M18** | 63.79 (16.69)  N=126 (146 missing) | 66.03 (16.78)  N= 157 (107 missing) |  |
| **Δ (M18-M0)** | -0.86 (13.09) | 0.69 (14.34) | -1.77 (-4.79; 1.25)  p= 0.25 |
| **M24** | 62.42 (17.23)  N= 110 (162 missing) | 65.93 (15.64)  N= 147 (117 missing) |  |
| **Δ (M24-M0)** | -1.44 (15.00) | 0.09 (12.94) | -2.21 (-5.39; 0.97)  p= 0.17 |

* Adjusted difference on the WHOQOL-PSY at M0 (the difference is in favour of the IG if it is positive).

III. WHOQOL social relationship

| **Criteria** | **INTERVENTION Group**  **Mean (standard deviation)**  **(N=272)** | **CONTROL Group**  **Mean (standard deviation)**  **(N=264)** | ***Adjusted difference**  **INTERVENTION– CONTROL**  **Mean (95% CI)** |
| --- | --- | --- | --- |
| **M0** | 69.17 (15.01)  N = 270 (2 missing) | 70.94 (16.30)  N= 264 (0 missing) |  |
| **M6** | 70.03 (15.68)  N=181 (91 missing) | 71.85 (15.28)  N=208 (56 missing) |  |
| **Δ (M6-M0)** | 1.00 (14.28) | 1.18 (14.99) | -0.88 (-3.42; 1.66)  p= 0.50 |
| **M12** | 72.54 (18.24)  N=159 (113 missing) | 73.65 (16.71)  N=191 (73 missing) |  |
| **Δ (M12-M0)** | 3.61 (18.31) | 2.75 (15.07) | 0.04 (-3.13; 3.21)  p= 0.98 |
| **M18** | 70.37 (17.34)  N=126 (146 missing) | 71.95 (17.86)  N= 157 (107 missing) |  |
| **Δ (M18-M0)** | 2.41 (16.07) | 1.83 (16.08) | -0.29 (-3.75; 3.16)  p= 0.87 |
| **M24** | 70.23 (16.49)  N= 110 (162 missing) | 72.28 (16.70)  N= 147 (117 missing) |  |
| **Δ (M24-M0)** | 2.08 (16.07) | 1.30 (16.55) | -0.59 (-4.14; 2.95)  p= 0.74 |

* Adjusted difference on the WHOQOL-SOCIAL at M0 (the difference is in favour of the IG if it is positive).

IV. WHOQOL environment

| **Criteria** | **INTERVENTION Group**  **Mean (standard deviation)**  **(N=272)** | **CONTROL Group**  **Mean (standard deviation)**  **(N=264)** | ***Adjusted difference**  **INTERVENTION– CONTROL**  **Mean (95% CI)** |
| --- | --- | --- | --- |
| **M0** | 76.36 (12.67)  N = 270 (2 missing) | 77.15 (13.18)  N= 264 (0 missing) |  |
| **M6** | 76.67 (12.69)  N=181 (91 missing) | 78.44 (11.59)  N=208 (56 missing) |  |
| **Δ (M6-M0)** | 0.73 (10.40) | 0.77 (10.90) | -0.76 (-2.66; 1.14)  p= 0.43 |
| **M12** | 80.09 (12.86)  N=159 (113 missing) | 78.58 (12.41)  N=191 (73 missing) |  |
| **Δ (M12-M0)** | 4.46 (12.17) | 0.57 (11.56) | 2.87 (0.62; 5.11)  p= 0.01 |
| **M18** | 77.48 (13.81)  N=126 (146 missing) | 79.06 (13.21)  N= 157 (107 missing) |  |
| **Δ (M18-M0)** | 1.37 (11.99) | 1.07 (11.81) | -0.38 (-3.00; 2.24)  p= 0.77 |
| **M24** | 75.97 (14.52)  N= 110 (162 missing) | 77.04 (12.77)  N= 147 (117 missing) |  |
| **Δ (M24-M0)** | 0.55 (12.54) | -1.51 (12.25) | 0.80 (-2.04; 3.65)  p= 0.58 |

* Adjusted difference on the WHOQOL-ENV at M0 (the difference is in favour of the IG if it is positive).

**DASHQ**

| **Criteria** | **INTERVENTION Group**  **Mean (standard deviation)**  **(N=272)** | **CONTROL Group**  **Mean (standard deviation)**  **(N=264)** | ***Adjusted difference**  **INTERVENTION– CONTROL**  **Mean (95% CI)** |
| --- | --- | --- | --- |
| **M0** | 42.74 (11.46)  N =270 (2 missing) | 41.05 (11.26)  N= 264 (0 missing) |  |
| **M6** | 43.03 (10.35)  N= 181 (91 missing) | 41.59 (10.86)  N= 208 (56 missing) |  |
| **Δ (M6-M0)** | -0.68 (8.59) | -0.11 (8.91) | 0.14 (-1.43; 1.71)  p= 0.86 |
| **M12** | 45.60 (10.92)  N=159 (113 missing) | 43.34 (11.33)  N=191 (73 missing) |  |
| **Δ (M12-M0)** | 1.51 (9.79) | 1.89 (9.00) | 0.50 (-1.33; 2.34)  p=0.60 |
| **M18** | 45.06 (10.02)  N= 126 (146 missing) | 42.05 (11.08)  N= 157 (107 missing) |  |
| **Δ (M18-M0)** | 0.51 (9.21) | 0.25 (9.31) | 1.29 (-0.71; 3.29)  p= 0.21 |
| **M24** | 45.65 (10.65)  N=110 (162 missing) | 43.04 (11.12)  N=147 (117 missing) |  |
| **Δ (M24-M0)** | 1.36 (9.96) | 1.08 (9.44) | 1.17 (-1.01; 3.36)  p= 0.29 |

* Adjusted difference on the DASHQ at M0 (the difference is in favour of the IG if it is positive).

**IPAQ**

| **Criteria** | **INTERVENTION Group**  **Mean (standard deviation)**  **(N=272)** | **CONTROL Group**  **Mean (standard deviation)**  **(N=264)** | ***Adjusted difference**  **INTERVENTION– CONTROL**  **Mean (95% CI)** |
| --- | --- | --- | --- |
| **M0** | 2453.20 (3059.36)  N =254 (5 missing) | 2390.00 (2707.07)  N= 257 (7 missing) |  |
| **M6** | 3677.00 (4431.90)  N= 179 (93 missing) | 2775.00 (3288.08)  N= 205 (59 missing) |  |
| **Δ (M6-M0)** | 1171.00 (3858.08) | 542.60 (3417.35) | 726.39 (22.91; 1429.88)  p= 0.04 |
| **M12** | 4150.9 (7886.25)  N= 154 (118 missing) | 3845 (8970.17)  N= 185 (79 missing) |  |
| **Δ (M12-M0)** | 1717.40 (8309.00) | 1541.00 (9271.44) | 292.47 (-1559.43; 2144.37)  p=0.76 |
| **M18** | 2781.00 (2842.79)  N= 125 (147 missing) | 2491.30 (3487.27)  N= 150 (114 missing) |  |
| **Δ (M18-M0)** | 506.90 (3327.39) | 269.30 (4452.39) | 273.34 (-503.89; 1050.57)  p= 0.49 |
| **M24** | 2191.80 (3553.58)  N= 104 (168 missing) | 1745.40 (2260.99)  N=138 (126 missing) |  |
| **Δ (M24-M0)** | -259.40 (4349.33) | -267.60 (3125.41) | 399.10 (-354.11; 1152.31)  p= 0.30 |

* Adjusted difference on the IPAQ at M0 (the difference is in favour of the IG if it is positive).

**BMI**

| **Criteria** | **INTERVENTION Group**  **Mean (standard deviation)**  **(N=272)** | **CONTROL Group**  **Mean (standard deviation)**  **(N=264)** | ***Adjusted difference**  **INTERVENTION– CONTROL**  **Mean (95% CI)** |
| --- | --- | --- | --- |
| **M0** | 27.42 (5.35)  N =270 (2 missing) | 26.70 (4.60)  N= 264 (0 missing) |  |
| **M6** | 27.59 (5.74)  N=181 (91 missing) | 26.56 (4.84)  N= 208 (56 missing) |  |
| **Δ (M6-M0)** | -0.10 (1.77) | -0.06 (1.01) | 0.03 (-0.32; 0.25)  p= 0.81 |
| **M12** | 27.05 (5.40)  N= 158 (114 missing) | 26.38 (4.63)  N= 189 (75 missing) |  |
| **Δ (M12-M0)** | -0.39 (1.46) | -0.18 (1.20) | -0.19 (-0.47; 0.10)  p=0.20 |
| **M18** | 27.39 (5.50)  N=126 (146 missing) | 26.60 (4.71)  N= 157 (107 missing) |  |
| **Δ (M18-M0)** | -0.05 (1.58) | 0.12 (1.41) | -0.15 (-0.50; 0.20)  p= 0.40 |
| **M24** | 27.05 (5.61)  N=110 (162 missing) | 26.71 (4.88)  N= 147 (117 missing) |  |
| **Δ (M24-M0)** | -0.29 (1.79) | 0.04 (1.41) | -0.32 (-0.71; 0.08)  p= 0.11 |

* Adjusted difference on the BMI at M0 (the difference is in favour of the IG if it is negative).

**ALCOHOL**

| **Criteria** | **INTERVENTION Group**  **Mean (standard deviation)**  **(N=272)** | **CONTROL Group**  **Mean (standard deviation)**  **(N=264)** | ***Adjusted difference**  **INTERVENTION– CONTROL**  **Mean (95% CI)** |
| --- | --- | --- | --- |
| **M0** | 1.30 (1.44)  N =270 (2 missing) | 1.46 (1.58)  N=264 (0 missing) |  |
| **M6** | 1.33 (1.15)  N= 181 (91 missing) | 1.47 (1.49)  N= 208 (56 missing) |  |
| **Δ (M6-M0)** | 0.13 (1.25) | 0.09 (1.41) | -0.05 (-0.28; 0.18)  p= 0.67 |
| **M12** | 1.23 (1.32)  N= 159 (113 missing) | 1.22 (1.18)  N=191 (73 missing) |  |
| **Δ (M12-M0)** | -0.038 (1.42) | -0.18 (1.46) | 0.06 (-0.17; 0.30)  p=0.60 |
| **M18** | 1.21 (1.37)  N= 126 (146 missing) | 1.16 (1.00)  N= 157 (107 missing) |  |
| **Δ (M18-M0)** | 0.02 (1.49) | -0.16 (1.38) | 0.09 (-0.17; 0.35)  p= 0.50 |
| **M24** | 1.12 (1.28)  N= 110 (162 missing) | 1.24 (0.95)  N= 147 (117 missing) |  |
| **Δ (M24-M0)** | 0.04 (1.24) | -0.07 (1.15) | -0.02 (-0.27; 0.23)  p= 0.89 |

* Adjusted difference on Alcohol at M0 (the difference is in favour of the IG if it is negative).
